# Are the Mineral Acids Formed in the Mouth?

**Published:** 1870-11

**Authors:** 


### ARTICLE IX.

### *Are the Mineral Acids Formed in the Mouth?*

By Professor Chase.

I propose to take the affirmative of the above proposition, and I shall attempt to show that the powerful mineral acids, sulphuric, nitric and hydrochloric, are formed in the mouth under certain circumstances; namely, when the food is allowed to remain around, upon and between the teeth day after day. They then are constantly being formed slowly, and in small quantities, to be sure. It is conceded by all who have given the subject any thought, I believe, that *organic* acids, such as *lactic*, *acetic*, etc., exist in the mouth, under certain conditions, in sufficient quantities to be detected. This is a well known fact and will not be denied, and it is not probable that they alone are the only acids which cause such disastrous results to the dental organs. It is true, the only positive evidence we have that nitric and sulphuric acids are ever present in the oral cavity, are the effects which they produce upon the teeth; but as for hydrochloric acid it has often been detected by reagents.

It seems to be a pretty well settled fact that dental decay is due to the action of certain acids, and if we knew to what acid or acids this action was due it would be an easy matter to apply the proper remedies.

I will now proceed to consider the action and formation of the different acids, and see if under any circumstances they could possibly be formed in the mouth. I will commence with

Sulphuric Acid. $H_2SO_4$. As its formula indicates it is composed by weight of two parts of hydrogen, sixty-four of oxygen and thirty-two of sulphur. A great number of the nitrogenous compounds, as albumen, caseine, gluten, &c., besides containing carbon, oxygen, hydrogen, and nitrogen in their composition, have a trace of sulphur. If for example a fibre of meat is caught between the teeth and allowed to remain there a sufficient length of time, it will undergo decomposition, a part of its hydrogen uniting with part of the nitrogen, forming ammonia, $NH_3$, its carbon uniting with part of oxygen forming carbonie acid $CO_2$, and the sulphur combining with the remaining hydrogen forming sulphuretted hydrogen, in contact with the oxygen of the air, is decomposed; the hydrogen uniting with the oxygen, forming water, and the sulphur set free. This sulphur being in the nascent state and having a great affinity for oxygen, is oxidized, and the result is *sulphurous acid* $SO_2$, which is converted rapidly into *sulphuric acid* in the presence of the water of the saliva; thus $SO_2+2H^2OH^2O_4S+2H$. The acid thus formed immediately acts upon the carbonate of lime, decomposing it, and charring the animal portion. This acid can neither decompose the phosphate of lime nor dissolve it. Therefore the latter, with the animal portion of the tooth, acts as a barrier, protecting the portion of tooth beneath it. It is probable that the "black variety" of decay, where but little of the tooth substance has been removed and the disintegrated portion is tough and cuts like leather, is caused by the slow but constant action of sulphuric acid. The acid blackens the organic or animal portion of a tooth by its great affinity for water, therefore it causes a portion of the oxygen, and hydrogen of the organic substance to unite, *forming water*, leaving an undue portion of carbon, which gives it its black or brown color. Owing to the small amount of sulphur which is present in the mouth under the most favorable circumstances, it necessarily follows that but a small quantity of sulphuric acid would be formed, and this immediately acting upon the teeth or some ingredient of saliva, may account for its never being discovered in the mouth in an uncombined state.

*Nitric Acid*— Symbol $HNO_3$, is, as its symbol indicates, composed by weight of one part of hydrogen, forty-eight parts of oxygen and fourteen parts of nitrogen; in one hundred parts we have hydrogen 1.59—, oxygen 77,19+, nitrogen 22.22+.

It is commonly prepared by the action of sulphuric acid upon potassium nitrate; the reaction is as follows: $KO_3N + H_2O_4S = KHO_4S + HNO_3$. Nitric acid is characterised by its great affinity for bases and for its property of furnishing oxygen in its nascent state to oxidizable substances. It acts energetically upon a tooth, decomposing the carbonate of lime, setting carbonic acid free and forming the nitrate of lime and water, thus: $CaO_3C + 2HNO_3 = Ca2NO_3 + CO_2 + H_2O$. It cannot decompose the phosphate of lime, but readily dissolves it. It decomposes and destroys the animal portion of the tooth.

Thus we see that nitric acid removes each constituent of the tooth as soon as it comes in contact with it, either by decomposing it or dissolving it. This powerful acid is sometimes administered as a medicine, as is also sulphuric and hydrochloric; but probably not sufficiently often to account for its effects which are constantly seen upon the teeth according to the observations of many practitioners. Watt says "It is the principal agent in the production of the "white decay.'" By the putrefactive decomposition of any nitrogenous substance ammonia is evolved. And although nitrogen has a very feeble affinity for oxygen, yet under certain circumstances it combines with it, and forms several different compounds; and is it not reasonable to suppose that when some albuminous substance is undergoing decomposition in the mouth some of the conditions are present for the formation of some oxide of nitrogen? Although this has never been demonstrated, yet I think it very probable, for hundreds of chemical combinations and decombinations are taking place throughout the organism every hour that could not take place outside the body except under entirely different circumstances; for instances, hydrochloric acid is found in the gastric juice; this must evidently have been formed in the body by the decomposition of some chloride, the chloride of sodium probably. Now to generate hydrochloric acid outside of the body by the decomposition of this salt requires a greater heat than that of the body. Suppose that an albuminous substance is decomposed and *nitric oxide* NO, is formed which is not improbable; this coming in contact with the oxygen in the saliva is immediately converted into *nitric trioxide* $N_2O_3$, and this in the presence of the water in the saliva is decomposed into nitric oxide and *nitric acid*. The reaction is as follows: $3N_2O_3+H_2O=2HNO_3+4NO$. Thus by the decomposition of a particle of meat or a portion of the gluten of flour, or of epithelium we may and probable do have formed one of the *strongest acids*. Watt says that the ammonia which is generated "exposed to the action of oxygen is *always* decomposed, an oxide of nitrogen being formed, and of course *nitric acid* is the result." Hydrochloric Acid. Symbol HCl., is as its name indicates, composed of hydrogen and chlorine. The action of this acid dilute upon the teeth is very energetic. It decomposes the carbonate of lime, forming the chloride of calcium and liberating the carbonic acid; the hydrogen of the acid unites with part of the oxygen, forming water, thus $CaO^{2}C+2HCl=CaCl_2+H_2OCO_2$. The phosphate of lime is not decomposed but is very soluble in the hydrochloric acid and in this way is removed from the tooth. If the acid is strong some of the organic portion of the tooth is also acted upon. The action of the acid up n the teeth may account for that variety of decay where the inorganic portion is removed and the animal portion remains.

Now the question arises, how is the acid formed in the mouth? As I said before, it has frequently been detected by means of reagents. The chlorides of sodium and potassium are always present in the saliva, and it is undoubtedly formed by the the decomposition of one of these salts, its chlorine combining with the hydrogen of the water; and thus the acid is formed. Again if there were different metals in the mouth, such as gold fillings and those of amalgam; or a gold plate, and plugs of some other metal or *vice*

*versa*, we would have galvanic currents which would readily decompose the water and the chlorides, setting the elements free, and each one would unite with the one for which st had the greatest affinity. Now, hydrogen being an *electro positive* element, and chlorine *electro negative*, their affinities for each other consequently great, they would unite, and the result would be hydrochloric acid.

The soluble chlorides are no doubt decomposed by other means which we may never be able to demonstrate. Probably no man would pretend to say just how every chemical change in the body took place. These are some of the mysteries which, it would seem, were not intended for us to know.

In this paper I do not wish to be understood as intimating that the decay of teeth is entirely due to the action of the mineral acids, far from it; but I do think they exert a great influence upon dental decay, and in some cases it may be that it is entirely due to their action. The action of the organic acids that are found in the mouth no doubt is about the same as the other acids, but much less rapid in their effects. —*Missouri Dental Journal.*

---

